# Comparison of body composition components and anaerobic performance parameters of elite male motorcycle speedway riders between pre- and post-competitive season

**DOI:** 10.3389/fphys.2023.1049237

**Published:** 2023-01-18

**Authors:** Kamil Michalik, Stefan Szczepan, Maciej Markowski, Marek Zatoń

**Affiliations:** ^1^ Department of Human Motor Skills, Faculty of Physical Education and Sport, Wroclaw University of Health and Sport Sciences, Wroclaw, Poland; ^2^ Department of Swimming, Faculty of Physical Education and Sport, Wroclaw University of Health and Sport Sciences, Wroclaw, Poland; ^3^ Faculty of Physical Education and Sport, Wroclaw University of Health and Sport Sciences, Wroclaw, Poland; ^4^ Department of Physiology and Biochemistry, Faculty of Physical Education and Sport, Wroclaw University of Health and Sport Sciences, Wroclaw, Poland

**Keywords:** motorcycle speedway racing, sport level, athletic performance, anaerobic capacity, body composition

## Abstract

The purpose of this study was to compare body composition components and anaerobic capacity indices in elite motorcycle speedway riders before and after the competitive season. This study included 12 volunteer male motorcycle speedway riders from the highest speedway league in Poland (PGE Speedway Ekstraliga) (age: 22.7 ± 6.0 years, body height: 171.3 ± 4.0 cm, body weight: 62.5 ± 3.1 kg). Before and after the competitive season, an assessment of body composition and Wingate test (WAnT) on a cycle ergometer with analysis of acute cardiorespiratory and biochemical responses was conducted. Sport level for all riders was defined by the number of heats won (WS), the total number of points scored in all heats including bonus points (PTS + B), and the percentage of heats won (%W). The motorcycle speedway riders participated in an average of 75.3 ± 15.0 total heats (HS) and obtained PTS + B equal 136.8 ± 48.2. The anaerobic performance, body composition, cardiorespiratory and biochemical responses did not changed after the competitive season. The significant statistical correlations were noted between the differences in the Fatigue Index during WAnT and total number of heats (r = −0.61) and with PTS + B (r = −0.58). Positively correlations were observed also between HS and differences: fat mass (FM) (r = 0.60) and percentage of fat mass (r = 0.61) (all *p* < 0.05). These findings reveal that the HS during the competitive season is related to the stabilization of anaerobic capacity in the WAnT. The HS is positively correlated with an increase in fat mass, and additional fat mass can adversely affect athletic performance in speedway.

## Introduction

Motorcycle speedway riders can take part in speedway league competitions on a motorcycle with a maximum engine capacity of 500 cm^3^ after 16 years of age. The motorcycle weighs a minimum of 77 kg, has one gear (no transmission) and is characterized by the absence of brakes. During a single heat, four riders are required to complete four laps around the track (length: about 260–425 m) in a counter-clockwise direction, which takes about 60 s. A speedway match in the Polish league consists of 15 heats and lasts from two to 3 hours. The competition is held with unified rules for all riders (Federation International Motorsport, 2020). The determinant of the individual sports level of riders is the Average Heat Score (AHS) (the total number of points scored divided by the number of heats ridden in the match). In traditional scoring, 3 points are awarded for winning the heat, 2 points for 2nd place, 1 point for 3rd place and no points for last place. The AHS of every riders is updated after each competition and is based on their performance in each match (Polish Motorsport Association, 2021). Every league in the world has an identical method of calculating the AHS. The speedway competitive season in the highest speedway league in Poland (PGE Speedway Ekstraliga) lasts six months (from April until September). The number of matches depends on the schedule of the competitive season. The main phase (match and rematch) of the season consists of fourteen rounds. After this phase is played, the knockout phase (play-off system) begins, where four teams advance to the semi-finals, the winners of which will advance to the finals. Eight teams compete in the PGE Speedway Ekstraliga (applies to the competition formula before 2022). In the context of maintaining high motor potential for several months, such a volume of starts poses a significant challenge for coaches and the riders.

The scientific research on motorcycle speedway racing is limited (Doggart et al., 2014; Doggart and Martin, 2014; Kusznir, 2015; Williamson, 2020), and few papers have focused on finding key physical characteristics that predicts the sports level of motorcycle speedway riders (Martin et al., 2014; Martin et al., 2019; Michalik et al., 2022). It has been established that there is no evidence of associations between heart rate and finishing position during a heat (Martin et al., 2014). In another study, Martin et al. (2019) analysed the physical profile of experienced and inexperienced motorcycle speedway riders based on anthropometric measurements including limb length and functional tests concluding that low body height and low body weight and balance are key physical characteristics of a competitive motorcycle speedway rider (Martin et al., 2019). In contrast, a recent study by Michalik et al. (2022) focused on comparing sport level, body composition, anaerobic capacity level, acute cardiorespiratory responses in maximal anaerobic effort, between junior riders (age 19.7 ± 1.1 years) and senior riders (age 29.7 ± 5.2 years). This study found that seniors had a 4% higher body mass index (BMI) and 20.5% higher body fat mass (%FM). In contrast, a 2.3% lower fatigue index (FI) in the Wingate test was found in juniors. In addition, body height correlated with all indicators of the seniors’ sports level (from r = −0.41 to −0.55). It was found that anthropometric traits, body height, lean body mass (LBM) and body surface area (BSA) were related to the sports level of riders. Therefore, it seems crucial to maintain appropriate levels of body weight and fat mass during the competitive season.

The timing and nature of muscle work during a roughly 60-s motorcycle speedway race indicates that energy is provided by the combined interaction of aerobic and anaerobic sources of ATP resynthesis (Gastin, 2001). During the training sessions, lactate concentrations of >12 mmol∙L^−1^ after completing four laps at maximum intensity (unpublished data). The cited study by Michalik et al. (2022) indicated that senior riders achieved higher acute responses during the Wingate test (WAnT), peak oxygen uptake (VO_2_peak) and carbon dioxide excretion (VCO_2_peak) compared to junior riders. This demonstrates the greater efficiency of energy production by the aerobic system in seniors (Price et al., 2014). Additional importance was also attributed to metabolic acidosis tolerance, gas exchange efficiency and the need to complete intense training sessions. The authors found significant, moderate associations in senior riders between post-workout hydrogen ion concentration (r = −0.38) and carbon dioxide partial pressure (r = −0.45) and the average number of points scored by a rider per heat (avPTS) (Michalik et al., 2022). This indicates, the importance of the ability to tolerate metabolic acidosis, buffering capacity and respiratory compensation (McKenna et al., 1997), which are important for delaying the onset of fatigue and the ability to maintain high muscle intensity (Wan et al., 2017). Thus, it seems reasonable to monitor the level of these indicators before, during and after the competitive season. Presumably, more frequent competitors can develop anaerobic capacity in this way. However, we are not aware of studies that would confirm this supposition.

The scarcity of research on the physical characteristics of professional motorcycle speedway riders prompts further exploration of factors that can help speedway riders and coaches optimize training and achieve the highest sport level (Martin et al., 2014). While data from single studies is useful, the nature of such one-off assessments does not provide information on the development of the body. There is a lack of scientific research to determine whether there are changes in the physical potential of motorcycle speedway riders during the competition season. Therefore, the purpose of this study was to compare the levels of body composition components, anaerobic capacity, peak cardiorespiratory responses and post-exercise blood gas changes and lactate concentrations in the Wingate test in elite male motorcycle speedway riders before and after the competitive season. The second objective was to identify the relationship between differences in the diagnosed key physical characteristics and the sports level of the riders during the competitive season, expressed by the number of heats won, the total number of points scored in all heats, the percentage of heats won and their derivatives. We assumed (H_0_) that there would be no changes in body composition and that the higher number of heats run would be a stimulus to develop the anaerobic capacity including, cardio-respiratory responses of the riders.

## Materials and methods

### Participants

This study involved 12 volunteer male motorcycle speedway riders (eight junior riders and four senior riders) from two clubs, which participated in the finals stage in Poland’s top speedway league (PGE Speedway Ekstraliga). Age: 22.7 ± 6.0 (95 %CI: 18.9–26.5, min—max: 17.5–34.8 years), height: 171.3 ± 4.0 (95 % CI: 168.8–173.8, min—max: 166.0–178.0 cm), weight: 62.5 ± 3.1 (95 % CI 60.6–64.4, min—max: 58.9–68.0 kg). All participants were considered highly competitive with one junior world champion, two Speedway Grand Prix participants and the rest of them were national champions and national representatives. The following inclusion criteria were used: a) contracted to a PGE Polish Speedway Ekstraliga club, b) a minimum of 1 season of experience in speedway competition, c) no contraindications to exercise testing, d) age over 16. In order to allow juniors to gain more sports experience, motorcycle speedway racing rules allows competition between juniors and seniors during matches. Therefore, despite the two age categories of riders, no division was used during further analyses. Participants were informed of the potential risks of the experiment, and all gave written informed consent to participate. Respondents were informed that they could withdraw at any time. There were no cancellations of participation. The participants voluntarily took part in the research project. This study was approved by Wroclaw University of Health and Sport Sciences Research Ethics Committee (6/2015) and conducted in accordance with the Declaration of Helsinki at the Exercise Testing Laboratory (certified PN - EN ISO 9001:2009).

### Study design

The research took place during one competitive season of the PGE Polish Speedway Ekstraliga. Research participants appeared in the laboratory twice to have their blood pressure measured, blood drawn to determine haematological parameters, anthropometric parameters measured and anaerobic capacity assessed with the anaerobic Wingate test, accompanied by analysis of expiratory gases and heart rate. The first session (pre-season testing) in the lab was performed in the spring two weeks before the start of competitive season. The second session (post-season testing) was performed in the autumn within two weeks after the competitive period. Both testing sessions separated 24 weeks. All sessions in the lab were conducted by the same riders and at the same time of day (8:00 a.m.–10:00 a.m.). Firstly, a blood sample was collected between 8:00 and 8:30 a.m. after an overnight fast. After that, a blood pressure measurement was performed after 10-min of seating. Next, a body composition analysis was conducted. The participants were asked to abstain from heavy exercise, alcohol and caffeine for 24-h preceding the lab visit.

### Haematological parameters

Capillary blood was collected from the fingertip before the exercise test at rest to determine morphotic parameters: haemoglobin (Hb) and haematocrit (Ht) concentrations using an ABX Micros OT.16 (Horiba Medical, Japan).

### Body composition and anthropometry

Body height and body weight were measured using a WPT 200 medical scale (RADWAG, Poland) and body composition was determined with a FUTREX analyser 6100/XL (Futrex Tech, Inc., Gaithersburg, MD, United States) based on the near-infrared spectrophotometry (NIRS) method. The probe was placed on the middle of the biceps brachia muscle or the dominant upper limb. All of anthropometry and body composition measurements were performed before exercise test. The NIRS method has been approved to determine the %FAT in humans (Fukuda et al., 2017). Total body fat percentage (%FM), fat mass (FM) and lean body mass (LBM) were assessed. Body surface area (BSA) was estimated by applying height and weight using the equation for men (Yu et al., 2010):

$$BSA=79.8106.{Height}^{0.7271}.{Weight}^{0.398}$$

### Anaerobic capacity

To assess anaerobic capacity, the WAnT was performed (Bar-Or, 1987). The test was conducted on an Ergomedic E894 cycle-ergometer (Monark, Sweden). The cycle-ergometer was calibrated before the pre- and post-season testing began. A warm-up was then performed according to the recommendations and consisted of five minutes cycling with two 5-s “all—out” sprints in third and fifth minute (Bar-Or, 1987). After the warm-up, the subjects remained seated on the cycle-ergometer for five minutes. The flywheel load (in kilograms) was 7.5% of the subject’s body weight. The effort lasted for thirty seconds, and the responsibility of the participant was to work at the maximum (possible) frequency of rotation to reach maximum power as quickly as possible and to maintain it for as long as possible. The test subjects were motivated by verbal encouragement to perform the test at the highest intensity possible. After the test, the subject remained on the cycle-ergometer for five minutes. The cycle-ergometer was controlled by a computer and MCE v.2.3 software (MCE, Poland). Peak power output (PPO), total work (Wtot), which were expressed per kilogram of body weight, and LBM were calculated. A fatigue index (FI) was also calculated.

### Cardio-respiratory responses analysis

During the Wingate assessment, the subjects breathed through a mask, and the exhaled air was analysed by a Quark b^2^ device (Cosmed, Milan, Italy). The apparatus was calibrated with atmospheric air and a gas mixture of composition: CO_2_—5%, O_2_—16% and N_2_—79% before the measurements began. Recording of respiratory parameters was done in each breath (breath-by-breath). Peak lung ventilation (VE_peak_), oxygen uptake (VO_2peak_), and carbon dioxide excretion (VCO_2peak_) were measured. The results were averaged every 10 s and converted to minute values to exclude erroneous breaths due to coughing, sighing, and swallowing. Reducing “noise” and artifacts can improve data interpretation. Heart rate (HR) was measured using an RS400 sport-tester (Polar Electro, Finland) during the Wingate test and recorded by Quark b^2^ analyser software. For HR, a 10-s average was used. The predicted maximum heart rate (HRmax_PRED_) was calculated from the formula (Tanaka et al., 2001): 208 ‐ 0.7 * age, where: age (years). On this basis, the percentage heart rate (%HR_PRED_) with respect to HRmax_PRED_ was calculated. Systolic and diastolic blood pressures were measured at rest after being seated for 10-min using an aneroid sphygmomanometer (Riester, Jungingen, Germany).

### Blood gasometry and lactate concentration

Capillary blood was drawn from the fingertip into heparinized capillaries at rest before the start of the test and at the third minute after the end of the test to determine acid-base balance: pH, partial pressure of oxygen (pO_2_), partial pressure of carbon dioxide (pCO_2_) and bicarbonate concentration ([HCO_3_^−^]) using a RapidLab 348 analyser (Bayer, Germany). Lactate concentration ([La^−^]) was also measured on a photomer (LP 400 Dr. Lange, Germany).

### Sports level - Total score per season

The research used rider classification lists published in the public domain of the organizer of the PGE Polish Speedway Ekstraliga (Speedway Ekstraliga classification lists, 2021). They consisted of the results of the riders, which were obtained during the analysed season. The result of the data collection process were numerical sets, including the following diagnostic parameters, which indicated the athletes’ sports level:

Heats Total (HS)—the number of heats in which the rider participated in the competitive season.

Wins Sum (WS)—the number of heats won by a rider.

Points (PTS)—the total number of points scored by a rider in all heats.

Points Sum + Bonus Points (PTS + B)—the total number of points (including bonus points) earned by a rider in all heats.

Based on the above results, the following parameters were calculated:

Percentage of Wins (%W)—the percentage of heats won (0%–100%) by an rider.

Heat Points Average (avPTS)—the average number of points scored by a rider per heat (0-3 pt).

Heat Points Average + Bonus (avPTS + B)—the average number of points (including bonus points) earned by a rider per heat (0-3 pt).

Note: a bonus point is awarded to a rider when he crosses the finish line directly behind his teammate and ahead of at least one rival. Bonus points are taken into account when calculating a rider’s average heat score (AHS) or average match score (AMS) (the total number of points scored divided by the number of the match). However, they are not included in the team’s total score. In each match, the number of heats in which a rider competes, depends on their age category. A junior rider must compete in a minimum of two heats; the minimum number of heats for senior riders is not regulated. A rider may start in a maximum of seven times in a single match. In addition, the last two heats (14th and 15th) (called nominated heats) are theoretically started by the riders with the highest points score in the match (although, the right of choice is held by the coach, who can nominate any rider from the line-up) (Federation International Motorsport, 2020).

### Training activity during the competitive season

An interview with head coaches revealed that during the competitive season (from the spring until the autumn), riders compete in about fourteen to eighteen matches a season. In total, a rider can run about 30–130 heats in a single season. In addition to matches, a motorcycle speedway rider performs six to eight specialized training on the track per month to achieve a competitive level of fitness. Each specialized track training is approximately two and a half hours. At the beginning of the specialized track training, in order to adjust the motorcycle to the surface, a free track ride is used. The specialized track training is dominated by interval efforts, with about five sessions of four, 30-s drives (two laps/half of a normal heat) with a break of less than 15 min. In total, one specialized track training session includes approximately 20 rides. Furthermore, several starts with rides to the first corner (about 40 m) are performed (alone and with other riders). Each rider stands in a different starting position (gate) each time to be prepared for match conditions. In addition, riders may spend time to better adjust their equipment. Beyond the specialized track training, riders spend 1–2 h on different types of motorsports e.g. enduro or motocross. During the match, there are two to seven heats lasting 60 s (in two hours of competition). One hour before each specialized track training session or match starts, the motorcycle speedway rider performs a series of stretching, relaxation, stimulation, and flexibility exercises according to their own routine. Most of the riders also perform exercises on reaction time to the starting signal using special tools (WittySEM, Microgate, Mahopac, United States) to stimulate the nervous system (duration about two-three times 40 s task). During the competitive season, complementing specialized track training session are combined sessions aimed at strength-based efforts (one, 45-min workout per week) and improving motor coordination (one, 45-min workout per week). An additional workout that combines elements of combat sports, such as kickboxing, is used to help prevent injuries during a crash (one, 45-min workout per week). Endurance efforts in the form of several kilometres of continuous running (one, 45-min treadmill-based workout per week) and cycling (one, 45-min cycle ergometer-based workout per week) are also used. The specialized track-based session technology is based on the standardized training methodology of motorcycle speedway racing (Żurek 2022). The workout protocols used to improve motor abilities created by the conditioning coaches are also based on training methodology in motorcycle speedway racing approved by Polish Motorsport Association (Żurek 2022). Verbal confirmation was received from the head coaches that the presented training protocol was performed by all riders during the conducted investigation.

### Statistics

The quantitative investigation involved a 4-dimensional approach (alpha, power, sample size, and effect size). The sample size was established *a priori* using G*Power 3.1 software (3.1.9.2, Kiel, Germany) (Faul et al., 2007), the expected effect size for comparison differences between means (*t*-test or/and Wilcoxon signed-rank test) was set at (Cohen’s d) 0.8 (large), and the α level was set at 0.05 and the power (1-β) was set at 0.8 (Cohen, 1992). The required total sample size was estimated to be 12 participants. IBM SPSS Statistics version 26 software package (IBM, Inc., Chicago, United States) was used to statistically process the data. Results are presented as arithmetic mean ± standard deviation ( $\bar{x}$ ±SD), 95% Confidence Interval (95% CI) and range (min-max). The Shapiro-Wilk test was used to assess the normality of the distribution of the studied characteristics. The Student’s t-test for dependent samples was used to evaluate differences in selected variables before and after the competitive season. Pearson’s linear correlation coefficient was calculated to examine the relationship between the covariates. The calculated threshold values of correlation coefficients of 0.1, 0.3, 0.5, 0.7, and 0.9 were interpreted as small, moderate, strong, very strong and extremely strong correlations, respectively (Hopkins et al., 2009). A *p* level of <0.05 was taken as statistically significant. Effect Size (ES) Cohen’s d was calculated to show practical effect, using the following criteria: 0.0 ≤ trivial <0.2, 0.2 ≤ small <0.5, 0.5 ≤ moderate <0.8, 0.8 ≤ large <1.3, ≥1.3 very large (Cohen, 1992).

## Results

Table 1 shows the parameters that determine the sports level of the tested motorcycle speedway riders during the competitive season.

**TABLE 1:** Season scores mean ± standard deviation, 95% CI and range (min-max) of riders determining the sport level of the tested athletes through scores obtained during all heats in the competitive season.

Variable	Season scores		
	Mean ± SD	(95% CI)	Range (min—max)
HS (n)	75.3 ± 15.0	65.8–84.8	49.0–104.0
WS (n)	21.6 ± 11.1	14.5–28.7	3.0–39.0
%W	27.1 ± 11.1	20.1–34.2	6.1–43.4
PTS (n)	122.0 ± 45.1	93.4–150.6	44.0–196.0
avPTS (0–3)	1.6 ± 0.3	1.4–1.8	0.9–2.0
PTS + B (n)	136.8 ± 48.2	106.1–167.4	55.0–213.0
avPTS + B (0–3)	1.8 ± 0.3	1.5–2.0	1.1–2.2

HS, heats sum; WS, wins sum; %W, percent of wins; PTS, points; avPTS, heat points average; PTS + B, points sum + bonus points; avPTS + B, heat points average plus bonus.

In tests performed after the competitive season, no significant differences were found between body composition components (Table 2), anaerobic capacity levels assessed by the Wingate test (Table 3), resting blood pressure, haematological parameters, cardiorespiratory responses measured during the Wingate test, post-exercise blood gasometry and lactate concentration (Table 4), compared to baseline tests performed before the start of competitive season.

**TABLE 2:** Pre- and post-season testing mean ± standard deviation and 95% CI of anthropometrics variables.

Variable	Pre-season testing		Post-season testing		Differences post-pre	
	Mean ± SD	(95% CI)	Mean ± SD	(95% CI)	Δ	%
Weight (kg)	62.5 ± 3.1	60.6–64.4	62.1 ± 1.9	61.0–63.4	−0.4	−0.6
BMI (kg∙m^−2^)	21.4 ± 1.1	20.7–22.0	21.1 ± 1.0	20.5–21.8	−0.3	−1.4
BSA (m^2^)	1.7 ± 0.1	1.7–1.8	1.7 ± 0.0	1.7–1.8	0.0	0.0
%FM (%)	10.2 ± 2.8	8.4–12.0	10.5 ± 2.5	8.8–12.1	0.3	2.9
FM (kg)	6.4 ± 2.0	5.2–7.7	6.5 ± 1.7	5.5–7.6	0.1	1.6
LBM (kg)	56.1 ± 2.3	54.6–57.5	55.7 ± 1.9	54.5–56.9	−0.4	−0.7

BMI, body mass index; BSA, body surface area, %FM, percentage of body fat in total body weight; FM, fat mass; LBM, lean body mass, Δ and % difference with respect to the pre-season status; *, statistically significant difference (*p* < 0.05).

**TABLE 3:** Pre- and post-season testing mean ± standard deviation and 95% CI of anaerobic performance determined in the Wingate test.

Variable	Pre-season testing		Post-season testing		Differences post-pre	
	Mean ± SD	(95% CI)	Mean ± SD	(95% CI)	Δ	%
PPO (W)	711.3 ± 78.5	661.5–761.2	704.4 ± 80.4	653.3–755.5	−6.9	−1.0
PPO/kg (W∙kg^−1^)	11.4 ± 0.8	10.8–11.9	11.3 ± 1.1	10.6–12.0	−0.1	−0.9
PPO/LBM (W∙LBM^−1^)	12.7 ± 1.2	11.9–13.4	12.7 ± 1.4	11.7–13.6	0	0.0
TW (kJ)	16.7 ± 1.4	15.8–17.7	16.5 ± 1.3	15.7–17.3	−0.2	−1.2
TW/kg (kJ∙kg^−1^)	267.6 ± 14.4	258.5–276.7	264.4 ± 16.8	253.8–275.1	−3.2	−1.2
TW/LBM (kJ∙LBM^−1^)	298.5 ± 21.0	285.1–311.8	295.6 ± 21.8	281.8–309.5	−2.9	−1.0
FI (%)	23.7 ± 3.6	21.4–26.0	24.3 ± 4.6	21.4–27.2	0.6	2.5

PPO, peak power output; PPO/kg, peak power output per body mass; PPO/LBM, peak power output per lean body mass; TW, total work; TW/kg, total work per body mass; TW/LBM, total work per lean body mass; FI, fatigue index; Δ and % difference with respect to the pre-season status; *, statistically significant difference (*p* < 0.05).

**TABLE 4:** Pre- and post-season testing mean ± standard deviation and 95% CI of cardio-respiratory responses, blood gas metrics and hematological variables, lactate ions concentrations measured at the Wingate test and blood pressure.

Variable	Pre-season testing		Post-season testing		Differences post-pre	
	Mean ± SD	(95% CI)	Mean ± SD	(95% CI)	Δ	%
VE_peak_ (l ∙ min^–1^)	131.5 ± 26.3	114.7–148.2	131.2±27.8	113.6–148.9	−0.3	−0.2
VE_peak_∙kg^–1^ (l ∙ min^–1^∙ kg^–1^)	2.1 ± 0.3	1.9–2.3	2.1±0.4	1.8–2.4	0	0.0
VE_peak_∙LBM^–1^ (l ∙ min^–1^∙ kg^–1^)	2.3 ± 0.4	2.1–2.6	2.4±0.5	2.0–2.7	0.1	4.3
VO_2peak_ (ml ∙ min^–1^)	2866.6 ± 506.7	2544.6–3188.5	2824.5±196.0	2700.0–2949.0	−42.1	−1.5
VO_2peak_∙kg^–1^ (ml ∙ min^–1^∙ kg^–1^)	45.8 ± 7.3	41.1–50.4	45.4±3.0	43.5–47.3	−0.4	−0.9
VCO_2peak_ (ml ∙ min^–1^)	3545.9 ± 674.9	3117.1–3974.7	3635.7±424.4	3366.0–3905.3	89.8	2.5
HR_peak_ (beats ∙ min^–1^)	186 ± 18	175–197	183±7	179–188	−2.9	−1.6
%HR_PRED_ (%)	97.0 ± 9.1	91.2–102.7	95.8±4.3	93.0–98.5	−1.2	−1.2
pH	7.22 ± 0.02	7.21–7.24	7.21±0.0	7.19–7.23	−0.01	−0.1
pO_2_ (mm Hg)	91.2 ± 5.2	87.9–94.5	88.4±8.6	83.0–93.9	−2.8	−3.1
pCO_2_ (mm Hg)	34.1 ± 3.6	31.8–36.4	34.8±5.5	31.3–38.3	0.7	2.1
[HCO_3_^-^] (mmol∙l–1)	14.8 ± 1.3	14.0–15.6	14.5±1.6	13.4–15.5	−0.3	−2.0
[La^-^] (mmol∙l–1)	11.5 ± 1.8	10.4–12.6	11.6±2.1	10.3–12.9	0.1	0.9
SP (mmHg)	127.6 ± 10.2	121.1–134.1	119.9±13.6	111.3–128.6	−7.7	−6.0
DP (mmHg)	72.7 ± 9.6	66.6-78.7	73.8±6.0	70.0–77.7	1.1	1.5
Hb (g∙dl^-1^)	14.7 ± 0.7	14.3-15.1	14.5±0.9	13.9–15.1	−0.2	−1.4
Ht (%)	42.7 ± 2.3	41.3-44.2	44.0±4.6	41.1–47.0	1.3	3.0

VE_peak_, peak minute ventilation; VE_peak_∙kg^−1^, peak minute ventilation per kilogram; VE_peak_∙LBM^−1^, peak minute ventilation pear lean body mass; VO_2peak_, peak oxygen uptake; VO_2peak_∙kg^−1^, peak oxygen uptake per kilogram; VCO_2peak_, peak carbon dioxide exertion; HR_peak_, peak heart rate; %HR_PRED_, percentage of predicted heart rate; pO_2_, oxygen partial pressure; pCO_2_, carbon dioxide partial pressure; [HCO_3_^−^], bicarbonate ions concentration; [La^−^], lactate ions concentration; SP, systolic blood pressure; DP, diastolic blood pressure; Hb, haemoglobin concentration; Ht, haematocrit; Δ and % difference with respect to the pre-season status; *, statistically significant difference (*p* < 0.05).

**FIGURE 1:**
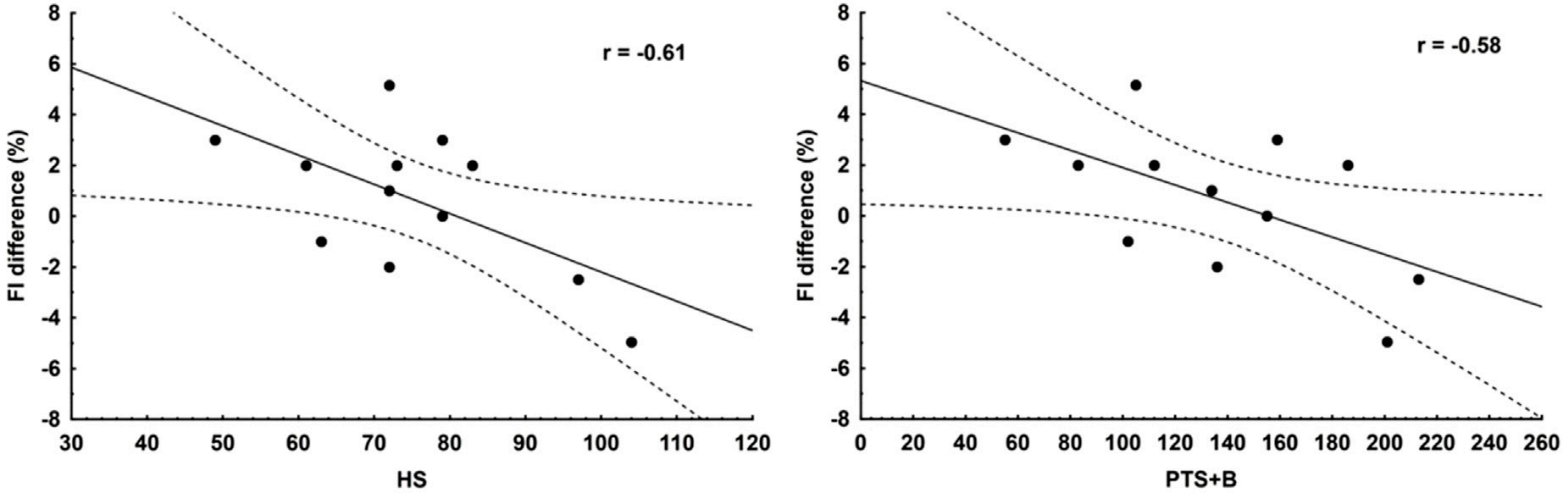
Correlation between the FI difference and the total number of heats in season (HS) and the total number of points (including bonus points) earned by competitors in all heats of the competitive season (PTS + B).

The FI difference statistically significantly correlated with HS r = −0.61 (*p* < 0.05) and PTS + B r = −0.58 (*p* < 0.05) (Figure 1). The correlation between the difference in FI and PTS was r = −0.56, which was on the borderline of the assumed level of statistical significance (*p* = 0.06). Strong correlations were also observed between HS and differences: FM r = 0.60 (*p* < 0.05) and %FM r = 0.61 (*p* < 0.05). There were not any significant differences between the components of body composition and anaerobic capacity.

## Discussion

In this investigation, the first objective was to verify whether pre- and post-competitive season key physical characteristics such as body composition, anaerobic capacity, peak cardiorespiratory responses, and post-exercise blood gasometry and lactate concentrations in the Wingate laboratory test would change in elite male motorcycle speedway riders. The second objective was to determine whether the sports level of riders, expressed as the total number of heats won, the total number of points scored in all heats, and their derivatives are related to changes in the previously mentioned physical characteristics. According to the data collected, from pre-season to post-competitive season, there were no changes in the components of body composition of motorcycle speedway riders. No significant changes were found in anaerobic capacity and ongoing cardiorespiratory and biochemical responses diagnosed during WAnT, which remained at similar levels. On the other hand, significant negative correlations (r = −0.61) were found between the number of heats (HS) or the total number of points plus bonus (PTS + B) scored in all heats during the competitive season (r = −0.58) and the FI difference in the Wingate test. In addition, the HS correlated positively with changes in body fat mass (r = 0.60) and %FM (r = 0.61).

In the sports science literature, there are examples of research that studied athletes before, during and after their competitive season, especially among representatives of team sports, such as: field hockey (Laurent et al., 2014), soccer (Emmonds et al., 2020), and basketball (Gonzalez et al., 2012). An interesting approach was used by Gonzalez et al. (2012), who compared seasonal performance changes according to the number of minutes spent playing basketball. In female athletes who played less frequently, there was no clear reduction in the level of strength and power, although the absolute values of the studied traits were higher in those who played more frequently (Gonzalez et al., 2012). This type of analysis was not common among representatives of motor sports, including motorcycle speedway racing. This is surprising, since evaluation conducted at regular intervals (e.g., pre- and post-season) allows monitoring of physical development and performance. The results presented in this paper are the first to report the outcomes of laboratory tests of motorcycle speedway riders between pre- and post-competitive season period. The number of heats and points earned by the riders participating in this study, two-thirds of whom were junior riders, was similar to the senior group presented in the article by Michalik et al. (2022). Therefore, this should be considered in the context of the lack of change in body composition components and the level of anaerobic capacity and cardiorespiratory responses.

In motorsports, the lower total mass of the riders and equipment reduces the athlete’s moment of inertia and inertial load, which, when generating the same level of power, positively affects the acceleration phase (Martin et al., 2019) and reaching top speed (Sánchez-Muñoz et al., 2011). Speedway motorcycles are restricted by regulations as to the minimum weight (77 kg), a lighter and smaller rider is desirable to take advantage of the potential of the engine power generated (Martin et al., 2019; Sánchez-Muñoz et al., 2011; D'Artibale et al., 2008). Consistent with the data, no changes in body composition components occurred in the riders between pre-season and post-season. The reported values are within the normal range of the athlete population, but special dietary interventions could be implemented to prevent unwanted fluctuations in body fat mass. The significant positive correlations between the number of heats and the changes in fat mass and %FM that were observed should be a warning in the long term. The human body requires the provision of adequate levels of energy to achieve and maintain energy balance, as a prerequisite for the maintenance of lean body mass (LBM) and immune function. This is particularly important in the development of optimal athletic performance (Clark et al., 2003). It should not be forgotten, that the exaggerated pursuit of weight reduction can consequently lead to a lower LBM. Therefore, regular monitoring during the season should reduce and minimize the potentially negative impact of body weight loss, which can have a negative impact on performance.

Motorcycle speedway racing provides a challenge for aerobic and anaerobic metabolism in providing energy to working muscles (Martin et al., 2014; Michalik et al., 2022). The demanding and precise muscle work during a race occurs with increasing levels of peripheral fatigue, which affects performance (Katakura et al., 2011). Therefore, the athlete must have the ability to tolerate changes in homeostasis and buffer increasing acidification to minimize fatigue (D'Artibale et al., 2008). The number of heats ridden might provide a stimulus to increase anaerobic performance, but pre- and post-season testing revealed no difference in this regard. A correlation analysis showed that a higher number of heats has a positive effect on the FI value measured in WAnT. The lack of differences in anaerobic capacity between junior and senior riders found in the work of Michalik et al. (2022), may be due to insufficient impact of training measures on the development of this trait. At the same time, it can be assumed that the anaerobic potential of senior riders is not developed to the maximum extent in the following years of training. The implication is that some riders have developmental reserves, and the application of appropriate anaerobic capacity training (in the motor aspect of strength and power) should raise exercise potential to a higher level.

Energy requirements during maximal, near 60-s exercise reflect the importance of anaerobic responses, although the aerobic system probably plays an equally important role. Previous research reported energy demands for individual sports such as 400 m and 400 m hurdles running (Zouhal et al., 2010) or 60-s simulated judo matches (Julio et al., 2017). In the Wingate test performed, there were no apparent adaptive changes in acute cardiorespiratory and biochemical responses from pre-season to post-season. In contrast, the previously reported by Michalik et al. (2022) differences between junior and senior riders in VO_2_peak and VCO_2_peak during WAnT may have been due to a difference of several years in the riders’ training seniority. Indeed, the role of the aerobic ATP resynthesis system is to ensure effective restitution between efforts (heats) (Tomlin and Wenger, 2001). In this study, the percentage of maximal heart rate clearly indicates that 30-s WAnT were performed near maximal intensity (twice above 95%). Similar results were reported in longer exercises, 96% and 94% for 60-s and 75-s tests, respectively (Carey and Richardson, 2003). In the present research, lower but non-significant resting systolic blood pressure (7.7 mm Hg) was reported during post-season testing. This hypotensive effect was found previous as post-exercise acute effect (Carpio-Rivera et al., 2016) and after regular training interventions de (de Barcelos et al., 2022). It could be caused by changes of blood volume, cardiac output and decreased total peripheral resistance (Joyner and Limberg, 2014). Additionally, de Oliveira et al. (2020) showed that lower resting SBP is related to better autonomic recovery what is important during intense training and/or competitive session. Therefore, further analyses of the physiological (and psychological) response of the body induced by a single training session and competition are needed. Tools for reporting subjective feelings of exertion, e.g., rating of perceived exertion (RPE) (Borg, 1982) or perceived readiness (PR) (Edwards et al., 2011), could also be included in this type of analysis. It is noteworthy that several weeks of training interventions (>2 weeks) such as high intensity interval sessions increase aerobic and anaerobic capacity (Boullosa et al., 2022).

There are limitations to this study. The current findings do not allow a direct cause and effect explanation of the observed lack of changes in body composition and anaerobic capacity of motorcycle speedway riders. First, the training measures used and participation in competitions can sustain the physical characteristics of the riders developed during the pre-season period. On the contrary, the lack of differences may also be due to the minimal impact of applied training loads during the competition period, unambiguously reporting insufficient physical (and motor) training for these riders. It would be ideal to utilize instrumentation to confirm the intensity during the specialized training sessions and competitions e.g. external load (distance, acceleration, velocity) and internal load, such as HR, RPE blood lactate concentration. Therefore, research should continue for several seasons to determine if there are individual development trends, stagnations or regressions in the physical characteristics of the riders. Second, the small number of motorcycle speedway riders examined did not allow the researchers to divide the athletes by junior and senior age categories. Furthermore, the anaerobic capacity of the upper limbs, as well as tests of upper and lower limb flexor and extensor force moments should be assessed. Subsequent examinations should include performing an assessment of body composition using the commonly used dual-energy X-ray absorptiometry (DXA). It seems no less interesting to study the changes in measurements taken during the pre-season period and the inclusion of tests during the competitive season, e.g., between the main and knockout phases. In addition to laboratory tests, field tests should be conducted during training sessions and/or competitions.

## Conclusion

Finally, the motorcycle speedway riders during their pre-season and post-season, revealed stabilization in the levels of body composition components, anaerobic capacity, cardiorespiratory responses, acid-based balance and blood lactate levels measured after the Wingate test. The high number of heats and the points achieved during the competitive season are related to the stabilization of anaerobic capacity, and impact the ability to maintain maximum power during WAnT expressed as fatigue index. The number of heats may influence an increase in fat mass. It is recommended that monitoring of body weight and body fat levels should be performed during the competition season. The data from this study should serve as a starting point for coaches to implement training methods that will develop the motorcycle speedway riders’ performance potential.

## Data Availability

Data cannot be shared publicly because of concerns over the risk of inadvertent disclosure of personal health information and performance information of high-level athletes. Data are available from the first author (contact via e-m: stefan.szczepan@awf.wroc.pl) for researchers who meet the criteria for access to confidential data. In order to proceed with permission to use the data set, it is necessary to conduct a joint investigation with the research staff.
